# Advances in Foodborne Pathogen Analysis

**DOI:** 10.3390/foods9111635

**Published:** 2020-11-10

**Authors:** Arun K. Bhunia, Bledar Bisha, Andrew G. Gehring, Byron F. Brehm-Stecher

**Affiliations:** 1 Department of Food Science, Purdue University, West Lafayette, IN 47907, USA; 2 Department of Comparative Pathobiology (Courtesy), Purdue University, West Lafayette, IN 47907, USA; 3 Department of Animal Science, University of Wyoming, Laramie, WY 82071, USA; 4 Molecular Characterization of Foodborne Pathogens, Agricultural Research Service, United States Department of Agriculture, Wyndmoor, PA 19038, USA; 5 Department of Food Science and Human Nutrition, Iowa State University, Ames, IA 50011, USA

**Keywords:** food safety, food pathogens, sample preparation, biosensors, rapid methods, detection, identification, food biosecurity

## Abstract

As the world population has grown, new demands on the production of foods have been met by increased efficiencies in production, from planting and harvesting to processing, packaging and distribution to retail locations. These efficiencies enable rapid intranational and global dissemination of foods, providing longer “face time” for products on retail shelves and allowing consumers to make healthy dietary choices year-round. However, our food production capabilities have outpaced the capacity of traditional detection methods to ensure our foods are safe. Traditional methods for culture-based detection and characterization of microorganisms are time-, labor- and, in some instances, space- and infrastructure-intensive, and are therefore not compatible with current (or future) production and processing realities. New and versatile detection methods requiring fewer overall resources (time, labor, space, equipment, cost, etc.) are needed to transform the throughput and safety dimensions of the food industry. Access to new, user-friendly, and point-of-care testing technologies may help expand the use and ease of testing, allowing stakeholders to leverage the data obtained to reduce their operating risk and health risks to the public. The papers in this Special Issue on “Advances in Foodborne Pathogen Analysis” address critical issues in rapid pathogen analysis, including preanalytical sample preparation, portable and field-capable test methods, the prevalence of antibiotic resistance in zoonotic pathogens and non-bacterial pathogens, such as viruses and protozoa.

Traditional enrichment culture combined with differential and selective plating serves as gold standards for the detection and identification of foodborne bacterial pathogens. Although accurate and sensitive, culture-based methods are labor-intensive and time consuming with the results often taking from days to weeks. There is a desire to replace these methods with “rapid methods” that typically employ highly sensitive and fast biosensor-based analysis [1]. However, creative thought and/or diligent empirical efforts are required to introduce paradigm shifts in the generation of rapid methods capable of replacing culture-based methods if the need for comparable sensitivity and accuracy is warranted. This Special Issue showcases contributions ranging from novel sample preparation techniques (e.g., advances in filtration, centrifugation, chemical or particle-based separation, etc.) for the selective, rapid, efficient, and/or quantitative recovery and concentration of targeted bacterial analytes to the development and/or improvement of rapid molecular detection and/or identification platforms (e.g., polymerase chain reaction (PCR), novel isothermal nucleic acid amplification techniques, matrix-assisted laser desorption/ionization time of flight mass spectrometry (MALDI-TOF MS), etc.). Key areas of research addressed in the special issue are detailed below, along with synopses of the relevant papers from the issue.

**Noroviruses:** Human noroviruses (HuNoVs) are the leading cause of acute gastroenteritis globally and, in the U.S., sicken ~685 million people annually [2]. Despite the outsize impact of HuNoVs on human health, relatively few researchers specialize in HuNoV biology, likely reflecting the difficulty and expense associated with establishing and conducting research in this field. Access to expertly curated information relevant to detection and analysis of HuNoVs may help expand this field and attract collaboration. Liu and Moore [3] provide a thorough survey of analytical techniques for noroviruses in which they compare existing methods, their salient characteristics and their relative advantages or limitations. Types of ligands that might be used for capture and concentration of noroviruses or to confer sensor specificity are reviewed in detail. Overall, the authors offer a valuable one-stop summary of issues critical to norovirus detection and analysis, including forward-looking considerations for sample preparation, that will benefit both novices and experts in the field.

**Preanalytical Sample Preparation:** Two papers in this issue address the issue of preanalytical sample preparation (“sample prep”), the various physical, chemical or other treatments needed to make a sample amenable to analysis. Quality of input into a detection step is critical to the overall success of the assay. While many papers may investigate individual sample prep methods, those that compare different approaches within the same study have a unique value. Armstrong et al. [4] applied a systematic approach to compare the utility of four different separation techniques (glass wool, 50 µm polypropylene filters, graphite felt, and continuous flow centrifugation (CFC)) for sample preparation. To define the physical effects associated with the use of these separation methods, a multifactorial analysis was performed where particle size and composition, both pre- and post-processing, were analyzed for four different food matrices including lean ground beef, ground pork, ground turkey and spinach. Retention of three important foodborne bacterial pathogens (*Escherichia coli* O157:H7, *Salmonella enterica*, and *Listeria monocytogenes*) was also examined to evaluate the value of these methods within the context of foodborne pathogen detection. Data from the multifactorial analysis not only delineated the particle size ranges but also defined the unique compositional profiles and quantified the bacterial retention. The three filtration membranes allowed for the passage of bacteria with minimal loss while CFC concentrated the inoculated bacteria. In addition, the deposition and therefore concentration of food matrix observed with CFC was considerably higher for meat samples than for spinach. However, filtration with glass wool prior to CFC helped clarify meat samples, which led to considerably lower amounts of post-processing solids in the CFC vessel and increased recovery of bacteria. Overall, these authors provide a framework for the deductive selection of appropriate sample preparation techniques, empowering the research community with knowledge of compatible sample prep/detection method pairings.

Biofilms, complex assemblages of microorganisms, polysaccharides, extracellular DNA and other environmental detritus, represent a special challenge for sample preparation and detection. Although most laboratories study bacteria in the free-swimming, planktonic form, biofilms are the dominant format for microbial life in nature. However, the inherent genotypic and physiological complexities of biofilms are barriers to their effective study and their presence on or within food processing equipment can serve as a recurrent source of product contamination. Aryal et al. [5] developed and optimized a microplate assay for generating multispecies biofilms consisting of *Listeria monocytogenes*, *Escherichia coli* O157:H7 and *Salmonella*, and also explored the use of hydrolytic enzymes for digestive detachment of these biofilms for downstream analysis. The authors found that trypsin was the most effective enzyme for biofilm detachment. Trypsin is routinely used for detachment of mammalian cells from culture vessel surfaces, suggesting its utility for biofilm detachment. Trypsin is used in volume in the pharmaceutical, silk and leather industries and is therefore widely available and relatively inexpensive. The work done here provides tools and knowledge that will help promote further understanding of mixed bacterial biofilms, including information relevant to their detection and inactivation.

**Nucleic Acid-Based Methods:** Nucleic acid-based methods for microbial detection and characterization have demonstrated their value over the past few decades. Despite a fairly long lag period between the invention of the polymerase chain reaction (PCR) in 1983 and its adoption by the food industry, it is now a firmly established and commercially supported method for the detection of foodborne pathogens. Resendiz-Nava and colleagues describe a nested PCR method for the detection of the protozoan pathogen *Cyclospora cayetanensis* in fresh berries and berry farm soils. Pushing the utility of this method beyond simple detection, the authors used Sanger sequencing of the resulting PCR products to enable phylogenetic analysis and attribution of geographical origin for *C. cayetanensis* isolates detected with the assay [6]. 

While PCR depends on temperature cycling, not all nucleic acid amplification methods do. Isothermal amplification methods belong to a broad family of nucleic acid amplification methods that do not depend on temperature cycling, but instead are carried out using static reaction temperatures. Independence from cycling obviates the need for expensive and potentially bulky thermal cycling instrumentation and potentially enhances the portability of isothermal methods. It is clear that the cells in our own bodies do not constantly cycle between extremes of 55 and 95 °C in order to replicate their DNA—this is done at body temperature, using enzymes to “melt” DNA and taking advantage of the reagent-rich milieu of the cytoplasmic gel architecture. Some isothermal amplification methods, such as Recombinase Polymerase Amplification (RPA), mimic the enzymatic processes, physical compartmentation and molecular crowding found in cellular DNA synthesis, with reaction optima of 37–42 °C—essentially “body temperature”. This latter property has practical implications for portability and utility under austere field conditions, allowing the use of simple heating equipment (including USB or solar sources), exothermic chemical reactions or the body’s own warmth to carry out reactions [7,8].

Two papers from the same author group used Recombinase Polymerase Amplification (RPA) for the detection of pathogens in foods. In the first paper, Li et al. developed an RPA assay targeting the conserved *Salmonella* fimbrial gene *fimY*, using a lateral flow (LF) readout for detection [9]. Although LF assays can generally be read qualitatively by eye (presence/absence), the authors used an LF test strip reader to generate quantitative results. The assay’s limit of detection for *Salmonella* in pure culture was 12 CFU/mL after a 10 min reaction time at 37 °C, and it could detect as low as 1.29 × 10^2^ CFU/mL in artificially contaminated vegetable samples. The assay was evaluated in parallel with the FDA-BAM method for *Salmonella* and a TaqMan-based qPCR assay developed in-house targeting the *fimY* gene, showing equivalence between the methods for naturally contaminated samples (seafood, chicken, vegetables) after enrichment.

In the second paper, Ma et al. expanded the *Salmonella* assay into a triplex *Staphylococcus aureus*/*Vibrio parahaemolyticus*/*Salmonella* test (15 min, 37 °C) and demonstrated its sensitivity, specificity and utility for screening natural samples processed according to the BAM methods for these pathogens [10].

**Zoonotic Dissemination of Antimicrobial Resistance (AMR): ***Enterococcus* spp. are ubiquitous in nature, and although foodborne transmission is rare, their intrinsic antibiotic resistance and their prevalence in the feedlot, beef processing, urban or surface water and clinical environments raises concern for the dissemination of AMR via horizontal gene transfer among these interactive biospheres [11]. New approaches for assessing the AMR threat from *Enterococcus* spp. are needed, especially those with the potential to be applied across the farm-to-fork-to-physician continuum. Anders and Bisha [12] evaluated the use of matrix-assisted laser desorption ionization time-of-flight mass spectrometry (MALDI-TOF MS) in conjunction with culture-based isolation for high-throughput detection and characterization of antimicrobial-resistant (AMR) enterococci from environmental sources (i.e., wildlife). *Enterococcus* spp. are recognized opportunistic pathogens, commonly found in gastrointestinal tracts of mammalian and avian species, which rapidly evolve to develop AMR genotypes and phenotypes, transferable to other bacteria, including several major bacterial pathogens. Wildlife are increasingly receiving attention as potential reservoirs of AMR bacteria and serve and vectors facilitating the dissemination of AMR. MALDI-TOF MS successfully identified 658 *Enterococcus* spp. isolates out of 718 presumptive isolates collected from gastrointestinal tracts of European starlings, which were captured near livestock operations in Colorado, Iowa, Kansas, Missouri, and Texas; antimicrobial susceptibility testing was then performed using 13 clinically significant antibiotics. While isolation and identification of target bacteria from the fecal material of wildlife is notoriously difficult, this work by Anders and Bisha provides ample evidence that MALDI-TOF MS can serve as a useful tool for high-throughput detection of enterococci in these types of samples.

In summary, this Special Issue on “Advances in Foodborne Pathogen Analysis” compiles eight papers addressing critical issues in rapid pathogen analysis, ranging from preanalytical sample preparation to new developments in nucleic acid-based testing, high-throughput screening across the production-to-consumption-to-disease continuum and studies related to non-bacterial pathogens, including viruses and protozoa. We thank the authors for their valuable contributions to this issue.
